# Assessment of chromium contamination in the soil and khat leaves (*Catha edulis Forsk*) and its health risks located in the vicinity of tannery industries; A case study in Bahir Dar City, Ethiopia

**DOI:** 10.1016/j.heliyon.2022.e11914

**Published:** 2022-12-01

**Authors:** Agegnehu Alemu, Alemwork Tegegne

**Affiliations:** a College of Science, Bahir Dar University, P.O Box 79, Bahir Dar, Ethiopia; b Blue Nile Water Institute, Bahir Dar University, Bahir Dar, Ethiopia

**Keywords:** Chromium, Khat leaves, Bioconcentration factor, Estimated daily intake, Target hazard quotient

## Abstract

Industrial effluents containing trace metals can contaminate water, soil and plants, as well as can cause serious human health impacts. The aim of this study was to assess the concentration of chromium in the soil and khat leaves using spectroscopic methods and to evaluate its potential human health risk on the consumers. The average concentrations of total chromium in the soil samples ranged from 71.01 ± 12.05 to 317.55 ± 23.14 mg kg^−1^. These values were greater than the control (7.6 ± 0.47 mg kg^−1^). The average concentrations of total Cr in the khat leaves ranged of 6.5 ± 1.76 to 30.01 ± 2.91 (mg kg^−1^). These values were higher than the maximum permissible Limits in vegetables 2.3 mg kg^−1^. The estimated daily intake (EDI) of an adult man weighing 70 kg and consuming on average 100 g of khat leaves per day was found in the range of 0.200–0.454 mg kg^−1^ body weight day^−1^. The target hazard quotient (THQ) of Cr from khat leaves consumption was found in the range of 0.001–0.076, which were <1, indicating no potential non-carcinogenic harmful health risk to the consumers in the society. Despite this, the regional EPA must pay close attention to controlling the use of irrigation water contaminated with tannery effluents in order to safeguard khat consumers. Furthermore, consumers must be aware of the health risks and refrain from consuming khat leaves cultivated in the study areas.

## 1 Introduction

Chromium (Cr) is a transition metal found in group VI B in the modern periodic table of the elements. It has a wide range of applications in both elemental and compound forms. In its elemental form, it is primarily used to make steel and alloys, i.e. to increase the metal strength and resistance to corrosion. Cr compounds are used for chrome plating, dye and pigment production, leather tanning agents, catalysis, wood treatment, foundry and laboratory chemical synthesis (Lunk, 2015).

The various industries that use Cr for different chemical processes generate a significant amount of Cr containing waste in to the environment. Cr may enter into the food chain through vegetation in dumping areas and plants irrigated by the untreated/semi treated wastewater from the tanning industries (Khan et al., 2013; Ugulu et al., 2021). Cr occurs in the environment mainly as Cr (III) and Cr (VI) oxidation states. Both chromium ions are interconvertible under favorable environmental conditions (pH, total Cr concentration, presence of reducing and oxidizing compounds, etc.) (Bokare and Choi, 2011). Cr (VI) is extremely toxic than Cr (III) and can cause cancer, damage liver, kidney, and skin of human beings (ATSDR, 2012). Cr (III) is essential in trace amounts for normal protein, fat, and carbohydrate metabolism (Zafra-Stone et al., 2007). It also helps for converting glucose into energy, encouraging healthy blood glucose and blood pressure levels.

Tanneries are pollution-intensive industries generating large amounts of complex liquid and solid wastes into the environment (Alemu et al., 2021; Verma and Sharma, 2022). Typically, Cr salts are used as tanning agents, with chromium sulfate accounting for about 90% of the tanning industry. Out of the total Cr applied in the tanning process, 30–40% remains as waste (Neelam et al., 2018). Most developing countries discharge tannery wastewater with little or no treatment into the water bodies & open lands (Hasnat et al., 2013). This is a common problem in Ethiopia. Studies indicate that Cr pollution in the water and sediments is increasing and becoming one of the main environmental concerns (Wosnie and Wondie, 2014; Alemu and Gabbiye, 2017).

**Figure 1:**
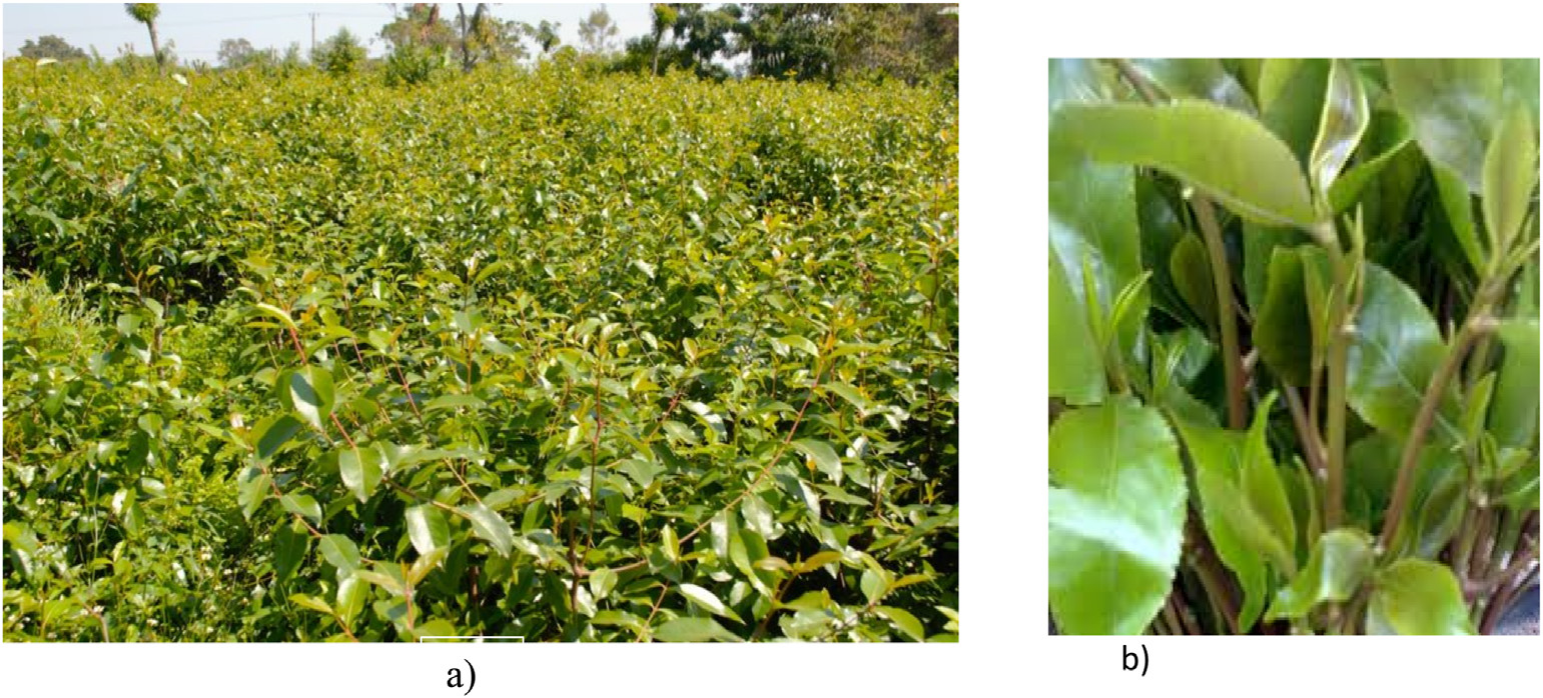
Khat plantation (a) and a bundle of khat leaves sold for consumption.

Khat (*Catha edulis Forsk*) is a flowering shrub with evergreen leaves that by many people chew for its stimulating effect (Woldamanuel, 2019). It grows well in parts of South Western Arabian Peninsula and East Africa (Odenwald et al., 2021). Khat producers in Africa include Ethiopia, Eretria, Somalia, Kenya, Zambia, Rwanda and South Africa. Beyond Africa, it is also produced in Arabian Peninsula, Yemen, Afghanistan, and Sir Lanka (Tadesse and Kebede, 2015). Khat is a source of income in these countries. Khat chewing is popular in some parts of Ethiopia, including Harar, Jimma, and Wlkite. It is also practiced in other parts of the country, such as Bahir Dar, Gondar, Debre Markos etc. Users chew approximately 100–500 g of khat leaves per day depending on its type and availability in the market (Gibbons and Arunotayanun, 2013). The khat plantation and a bundle of khat leaves sold in the market are shown in Figure 1(a) and (b) below.

Bahir Dar is one of the most beautiful cities in Ethiopia, which is established on the shores of Lake Tana and Abbay River. It is a hub of commercial and some industrial activities. The majority of industries lack appropriate wastewater treatment plants. Tanneries generated a large amount of Cr containing wastewater, which can be absorbed and accumulated in different parts of the khat plant species. As a result, Cr can enter the food chain and pose a risk of causing serious human health problems such as cancer (Hossain et al., 2017). Plants can absorb Cr from tannery waste via essential ion carriers such as sulfate (Kim et al., 2006). Cr uptake, accumulation, and translocation, depend on its speciation. Moreover, it also varies from one plant species to another (Ertani et al., 2017; Alemu et al., 2020).

The main objective of this study was to determine the concentration of Cr both in the soil (field) and khat leaves, which were irrigated from a small water canal that sometimes mixed with the effluents generated from the nearby tannery industries. Moreover, it investigated the bioconcentration factor of Cr in the khat leaves and the potential health risks on the khat consumers in the society.

## 2 Materials and methods

### 2.1 Description of the study area

The study area is found close to Bahir Dar City. The city is located in the northwest of Ethiopia at about 11°36′0″ N latitude and 37°24′0″ E longitude at an elevation of 1800 m. It has warm climate in the winter and high rainfall in the summer (June 25–September 25). The study area covers a water canal used by local farmers to irrigate khat plants as well as vegetables and fruits. The canal runs under the drainage lines from two tannery industries and there are some occasions that the tannery wastewater mixes with the water canal.

### 2.2 Sampling sites

**Figure 2:**
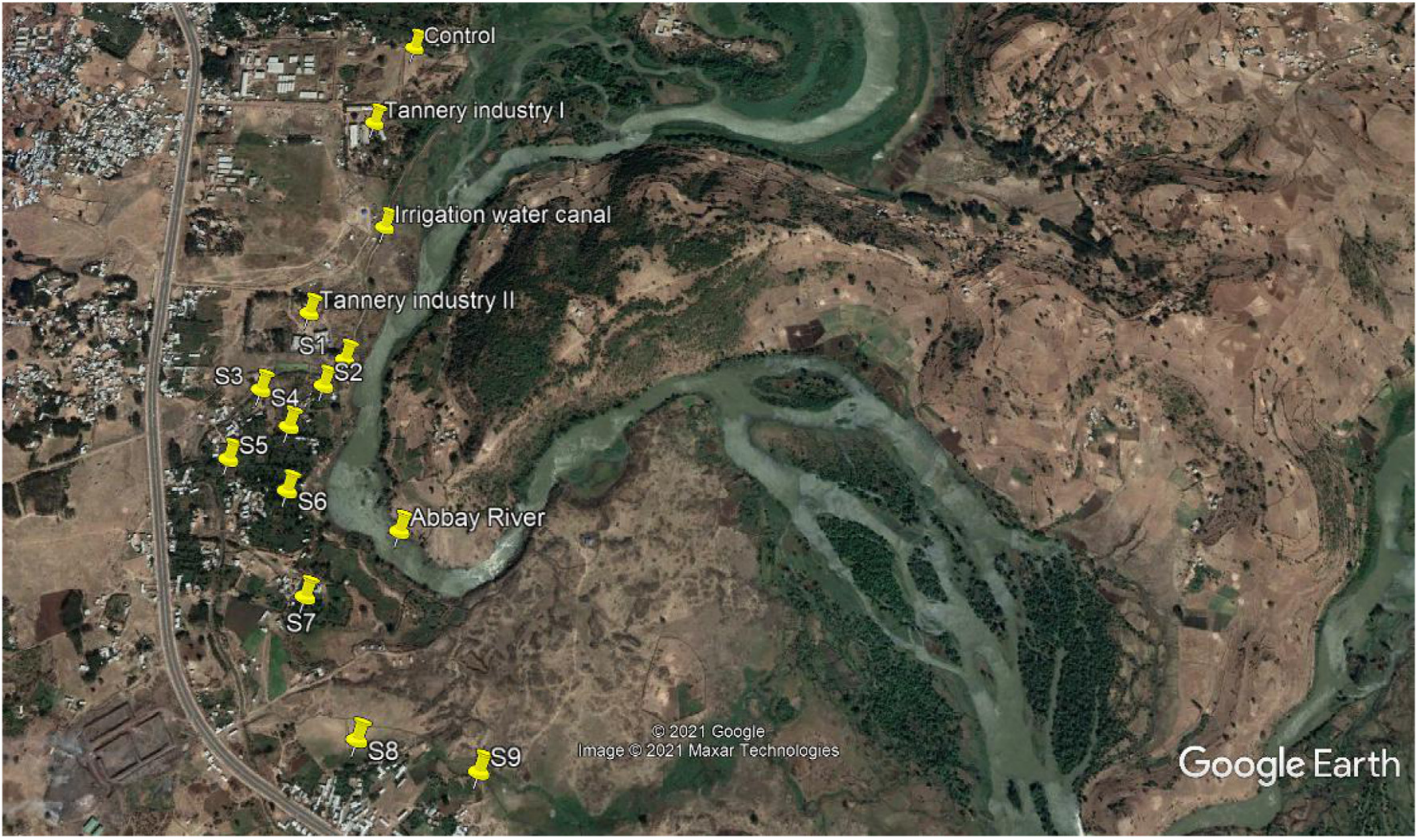
Location of sampling sites from S1 to S9 along the water canal (Google Earth).

Khat leaves and soil samples were collected randomly in the farm lands from 9 sites (S1–S9) along the water canal of khat plantation (Figure 2). The control site (C) was chosen above the tannery industries in the upstream of Abbay River. The sampling sites were chosen randomly to assess the level of contamination of Cr both in the Khat leaves and soil samples. S1 is found at about 0.5 km from the second tannery and S9 is found close to Sevatamit, which is a satellite city of Bahir Dar.

### 2.3 Reagents and instruments

All chemicals and reagents used were of analytical grade. KCl (99.9%, Fisher Scientific), 1, 5-diphenylcarbazide (assay ≥99%, Merck), acetone (assay 99.5%, Royal chemicals), Na_2_CO_3_ (99.5%, Krishna Chemicals), KH_2_PO_4_ (99%, Merck), HNO_3_ (assay 68–70%) and H_2_O_2_ (30%) from BDH laboratory supplies.

### 2.4 Sample collection and preservation

Soil samples were collected in khat agricultural areas at about 5 cm depth below the surface by composite sampling system using clean polyethylene plastic bags. The samples were air-dried, ground using mortar and pistil and sieved through 250 μm mesh. It was then stored at 4 °C for digestion and analysis.

Khat leave samples were collected from the Khat plant farmlands that were identical to soil sampling sites. The khat leaf samples were cut and collected in a clean plastic bag. It was dried in an open air for one week and kept in an oven at 105 °C overnight. The dried khat leaves were powdered with mortar and pistil, and sieved through 250 μm diameter mesh. Finally, the powdered khat leaves were kept in a cleaned and labeled airtight Ziploc plastic bags for digestion and further analysis. of Cr.

### 2.5 Analysis of soil and plant samples

#### 2.5.1 Determination of soil pH

The pH of the soil samples was determined by mixing 10 g of dry weight soil with 25 mL of 1M KCl solution in the ratio of 1:2.5 (Kabala et al., 2016). It was stirred for 30 min, and the suspensions were measured with a pH meter (Hanna, HI8424).

#### 2.5.2 Determination of Cr (VI) and total Cr in the soil

The total Cr in the soil was determined by mixing 1 g of dry weight soil with 10 mL of 1:1 nitric acid in 250 mL flask using standard procedures (USEPA, 1996; Ugulu et al., 2021). The flask was covered with watch glass during digestion. It was heated to 95 ± 5 °C and then refluxed for 15 min. 5 mL of concentrated nitric acid was added and refluxed for 30 min. This procedure was repeated until the brown fumes in the sample were completely removed. 3 mL of 30% H_2_O_2_ and 2 mL of water were added to the flask and heated in the above temperature ranges until no effervescence was observed. The digestate was diluted and filtered using whatman filter paper. Finally, it was measured by using ICP-OES (Perkin Elmer Optima 8000).

Cr can exist in the soil in the form of Cr (VI). Extraction of Cr (VI) in the soil was done by following the procedure used by different researchers (Scancar et al., 2007; Lesniewska et al., 2017). 5 g of air-dried soil were transferred to 50 mL 0.005 M KH_2_PO_4_ and 0.05 M K_2_HPO_4_, mixed using rotary shaker (Mcl-300) at 200 rpm for 24 h. The mixture was filtered through whatman filter paper grade 42. The soil sample extracts were analyzed using PerkinElmer Lambda 3000 spectrophotometer with 1.5 Diphenylcarbazide at 540 nm (APHA, 1998).

#### 2.5.3 Determination of total Cr in Khat leaves

0.5 g of ground khat sieved in a 250 μm, 6 mL conc. HNO_3_, and 2 mL H_2_O_2_ were placed in a Teflon tube and closed tightly, digest for 30 min in a microwave digester at temperature of 200 °C and power 1200 w (Wu et al., 1997). It was cooled, filtered and transferred to 50 mL volumetric flask and diluted to the mark with distilled water. In deed the total Cr was determined with ICP-OES (PerkinElmer Optima 8000).

### 2.6 Bioconcentration factor (BCF)

BCF is the ratio of Cr concentration in edible part of khat leave to Cr concentration in the soil sample (Alemu et al., 2020). The transfer of Cr from the soil to the plant leaves is calculated by Eq. (1).

(1) $$BCF=\frac{C_{plant}}{C_{soil}}$$

where C_plant_ is the Cr concentration in the khat leave and C_soil_ is the Cr concentration in the soil.

### 2.7 Estimated daily intake (EDI) and target hazard quotient (THQ)

#### 2.7.1 Estimated daily intake (EDI) of Cr

The estimated daily intake (EDI) of Cr in the khat leaves was determined using Eq. (2) (Chary et al., 2008).

(2) $$EDI=\frac{C_{m}\times I_{g}}{B_{W}}$$

where: $C_{m}$ is the Cr concentration in khat leaves (mg kg^−1^ dry weight); $I_{g}$ is ingestion rate, which is taken as 0.1 kg day^−1^ (the smallest ingestion rate) for khat fresh leaves (Gibbons and Arunotayanun, 2013); $B_{w}$ is the average body weight of an adult person which is taken as 70 kg.

#### 2.7.2 Target hazard quotient (THQ)

The health risks of consumers from khat chewing irrigated with contaminated tannery wastewater was calculated using THQs Eqs. (2) and (3) (FAO/WHO, 2011). The non-carcinogenic health risks of khat consumers can be calculated using Eq. (3).

(3) $$THQ=\frac{E_{f}\times E_{D}\times F_{IR}\times C_{M}\times C_{f\;}}{R_{fD}{\times B}_{W}{\times T}_{A}}\times 0.001$$

where E_f_ is exposure frequency (365 day/year); ED is the exposure duration (65 years), F_IR_ is the average consumption of khat (100 g/person/day), CM is metal concentration (mg/kg dry weight); Cf is concentration conversion factor for fresh khat weight to dry weight (0.25) (Staven et al. 2003). $R_{fD}$ is the oral reference dose for the metal (mg kg^−1^ of body weight per day), Cr (1.5 mg kg^−1^ per day) (USEPA, 2015). BW is reference body weight for an adult, which is 70 kg. TA is the average exposure time (65 years × 365 days/years).

### 2.8 Percent of recovery (% R)

Percent of recovery was determined by adding known concentration of Cr to the soil and khat leave samples during the process of digestion. The concentrations of total Cr were analyzed for the spiked and unspiked samples. % R was calculated using Eq. (4) (APHA, 1998):

(4) $$\%\;R=\frac{Spiked-Unspiked}{Conc.\;of\;analyte\;added\;to\;the\;spike}\times 100$$

### 2.9 Statistical data analysis

Microsoft Excel and Origin lab software were used to analyze the collected data. One-way ANOVA at 95% confidence interval (p < 0.05) was used to determine the significant difference in mean concentration of total Cr in the soil and khat leaves in the sampling sites. Statistical analyses were done using SPSS Statistics 24.0.

## 3 Results and discussion

### 3.1 Quality control

The quality of data was controlled by using of standard procedures, calibrating with standard solutions, analysing of reagent blanks, and calculating the percent of recovery. The analyses were done in triplicate, and the average values were expressed as the mean ± SD. The calibration curve correlation coefficients (R) for the analysis of Cr in the soil and khat leaves using ICP-OES and UV-Vis spectroscopy were in the range of 0.989–0.999. The percentage of recovery tests for the soil and khat leaves were found 90% and 87%, respectively. This value falls within the acceptable range of 85–115% (USEPA, 1994).

### 3.2 pH of the soil samples

The pH of the soil samples was found in the range of 4.84 ± 0.18 to 6.1 ± 0.42 as shown in Table 1. The lowest pH value was shown at site S6 and S8. This suggests that the two sites have a high negative charge on the sediment/soil component (Jordao et al., 1997). Cr occurs in a stable form as Cr (III) under reduced conditions in the pH ranges of 4–8. Cr (III) oxidizes to Cr (VI) and vice versa in the presence of oxidizing and reducing agents, respectively (Avudainayagam et al., 2003). The acidic nature of the soil might be due to use of acidic salts in the processing of leather during the time of sampling. Cr (VI) occurs in an anionic form in most natural environments, predominantly as hydrogen chromate (HCrO_4_^−^) between pH 1 and 6. According to USDA (1993) Natural Resources Conservation Service, soil pH ranges roughly from acidic (pH 3.5) to very strongly alkaline (pH 9.0). Soil pH is an important indicator of soil health. It affects crop yields, crop suitability, plant nutrient availability, and soil micro-organism activity, influencing key soil processes. In this study, the average pH values were 5.48, which is in the range of USDA standard (Alemu and Gabbiye, 2017). This value was also equivalent to the control pH value (5.45 ± 0.05).

**Table 1:** pH of the soil samples.

Sample Code	Average pH
C	5.45 ± 0.05
S1	5.3 ± 0.07
S2	5.3 ± 0.07
S3	5.64 ± 0. 13
S4	5.78 ± 0.06
S5	5.7 ± 0.85
S6	4.84 ± 0.35
S7	5.76 ± 0.26
S8	4.84 ± 0.18
S9	6.1 ± 0.42

### 3.3 Level of Cr in the soil

The concentrations of Cr (VI) and total Cr in the soil samples collected are shown in Table 2 below. The level of Cr (VI) contamination of the soil samples was found in the range of 0.03 ± 0.01 to 0.1 ± 0.04 mg kg^−1.^ Higher total Cr concentrations were observed at site S9 (317.55 ± 23.14 mg kg^−1^) and S8 (278.85 ± 15.13 mg kg^−1^). The lowest value of total Cr was observed at S4 (71.01 ± 12.05 mg kg^−1^). All of the Cr (VI) and total Cr values were higher than the control and lower than the Ethiopian Environmental Quality Standards for soil (EEPA, 2003).

**Table 2:** Cr (VI) and Cr total concentration in the soil samples.

Sample code	Cr (VI) (mg kg−1)	Total Cr (mg kg^−1^)
C	ND	7.6 ± 0.47
S1	0.07 ± 0.05	157.31 ± 11.01
S2	0.07 ± 0.02	140.12 ± 8.13
S3	0.1 ± 0.04	186.68 ± 5.03
S4	0.04 ± 0.01	71.01 ± 12.05
S5	0.03 ± 0.01	128.67 ± 15.13
S6	0.05 ± 0.04	224.31 ± 13.39
S7	0.07 ± 0.03	182.54 ± 9.17
S8	0.07 ± o.o5	278.85 ± 15.13
S9	0.04 ± 0.04	317.55 ± 23.14

ND – Not detected.

According to single factor ANOVA, the average total Cr concentrations in the soil samples show significant variations, P < 0.05 at the sampling sites. This might be due to discharge of Cr containing wastewater from tannery industries found in the vicinity of the channel. The results were compared with other studies. In Kasur District (Pakistan), the Cr concentration in the soil was found up to 839 mg kg^−1^ from the vicinity of a tanning effluent and 1829 mg kg^−1^ in the area of old stagnant pool, which are much higher than the safe limits (Khan et al., 2013).

In Ethiopia, the levels of total Cr and Cr (VI) accumulation in the agricultural soil found in the range of 254.25–1581.66 and 1.17–2.23 mg/kg around the Ethiopia Tannery Share Company, respectively (Homa et al., 2016). Reda (2015) also reported a total Cr level of 2017.24 mg/kg in the surrounding soil of Modjo Tannery effluent. A study of Cr pollution of the soil in the riparian of Abbay River from tannery and textile industries indicated a maximum of an average value of 232.465 ± 56.219 mg/kg (Alemu and Gabbiye, 2017). The increased concentration of Cr in the soil can lead to an increase in the uptake and accumulation of Cr in the khat leaves that can affect the heath of the consumers.

### 3.4 Total Cr in khat (*Catha edulis Forsk*) leaves

The average concentrations of total Cr in khat leave were found in the range of 6.5 ± 1.76 to 35.71 ± 0.98 (mg kg^−1^). The highest total Cr concentration (35.71 ± 0.98 mg kg^−1^) was observed at S2 and the lowest total Cr (6.5 ± 1.76) was observed at PS4 (Table 3). There was a statistically significant difference (P < 0.05) in the mean concentration of total Cr in the khat leaves taken at different sampling sites. This might be variation in the distances of the sampling sites from the irrigation canal contaminated with the tannery wastewater. The total Cr concentrations in the khat leaves were higher than the maximum permissible Limits in vegetables, 2.3 mg kg^−1^ (FAO/WHO Codex Alimentarius Commission, 2001).

**Table 3:** Total Cr in the Khat leaves.

Sample ID	Total Cr (mg kg^−1^)
C	7.50
KS1	31 ± 1.32
KS2	35.71 ± 0.98
KS3	16 ± 1.01
KS4	6.5 ± 1.76
KS5	13 ± 0.87
KS6	28.08 ± 2.79
KS7	30.01 ± 2.91
KS8	27.75 ± 4.85
KS9	29.02 ± 3.22

NB: KS means Khat plant at sampling site

This study was compared to other similar studies in Eastern Ethiopia vegetables including khat cultivated by irrigating with sewage wastewater. The total Cr concentration in the khat leaves was found in the range of 9.04–15.54 mg kg^−1^ (Deribachew et al., 2015). Another study in the southern region of Ethiopia indicated that the total Cr (mg kg^−1^) in the khat leaves in Koyra, Didole, and Konso were 1.52 ± 0.13, 2.75 ± 0.32, and 4.09 ± 0.29 respectively (Desta and Ataklti, 2015). Maleki and Zarasvand (2008) reported 7.9 mg kg^−1^ of total Cr in vegetable samples.

### 3.5 Bioconcentration factor (BCF)

BCF provides quantitative information for assessment the risk of Cr from khat chewing to human health. BCF helps to estimate the plants potential to accumulate Cr in its tissue, especially in the edible part of the leaves of khat plant. The BCF value was found in the range of 0.086–0.255 at all sampling sites (Table 4). The calculated BCF values are less than one, which indicates the khat plant accumulates less Cr in its leaves (Baker, 1981). The BCF of Cr in tomato and cabbage at Koka and Ejersa, Ethiopia indicated 0.018 and 0.090, respectively (Bayissa and Gebeyehu, 2021). The BCF of Cr in the fruits of medicinal plants was found in the range of 0.3–0.439 (Parveen et al., 2020). Chang et al. (2014) also reported that the BCF of Cr in six leaf vegetables in the range of 0.0002–0.027. The BCF of Cr in this study was higher due to irrigation of contaminated tannery wastewater to the khat plants.

**Table 4:** Chromium BCF in the khat leaves.

Sample code	BCF
C	0.197
S1	0.197
S2	0.255
S3	0.086
S4	0.092
S5	0.101
S6	0.125
S7	0.164
S8	0.099
S9	0.091

### 3.6 Estimated daily intake (EDI) and target hazard quotient (THQ)

#### 3.6.1 Estimated daily intake (EDI)

The daily intake of Cr depends on its concentration in the khat leaves and its daily consumption of khat leaves. The effect Cr on humans can be influenced by an individual’s body weight. The EDI of an adult man with 70 kg weight who consumes on average 100 g of khat leaves per day in the study area was calculated and found in the range of 0.200–0.454 mg/kg body weight/day (Table 5). This value (EDI of Cr) will be increased for people with lower body weight and frequently consuming khat. The EDI of the control is 0.011 mg/kg body weight/day, which is very low compared to other sampling sites. The EDI of Cr for an adult person consuming 240 g/day of tomato and cabbage in Koka area, Ethiopia reported values of 2.76 × 10^−4^ and 1.52 × 10^−3^ mg/day/kg body weight, respectively (Bayissa and Gebeyehu, 2021). The daily intake of Cr in khat leaves was higher than the total intake from food (0.008 mg/day) (Shaheen et al., 2016).

**Table 5:** Calculated EDI and THQ values of Cr via chewing of khat.

Sample code	EDI (mg/kg. day)	THQ
C	0.011	0.002
S1	0.225	0.038
S2	0.200	0.034
S3	0.267	0.045
S4	0.102	0.001
S5	0.184	0.031
S6	0.321	0.054
S7	0.261	0.044
S8	0.399	0.067
S9	0.454	0.076

#### 3.6.2 Target hazard quotient (THQ)

The THQ of total Cr from the consumption of khat leaves in the study area found in the range of 0.001 (S4) to 0.076 (S9) (Table 5). In the study area, the calculated THQ values of Cr were less than 1.0. According to USEPA (2015), THQ >1 indicates a higher possibility to occur for non-cancer health disorders. The THQ values indicated that the level of Cr in the sampling sites were within the tolerable limit of non-carcinogenic harmful health risk in the study area. This doesn’t mean that all the consumers are safe from the risk of Cr gained through khat chewing. The THQ value can be varied based on the dose of khat leave consumption and body weight of the consumer.

## 4 Conclusions

This study found Cr concentrations in the soil and khat leaves grown using irrigation water contaminated with tannery wastewater and consumed by many people in Bahir Dar and the surrounding area. Furthermore, BCF, EDI and THQ health risk implications of Cr from chewing of khat leaves were investigated. The total Cr concentrations were found in the average range of 71.01 ± 12.05 to 317.55 ± 23.14 mg/kg in the soil and 6.5 ± 1.76 to 35.71 ± 0.98 mg/kg in the khat leaves. The total Cr concentrations in the khat leaves exceeded the maximum permissible Limit in vegetables (i.e. 2.3 mg kg^−1^). The EDI of Cr from consumption of khat leaves for an adult man (70 kg) on average was 0.268 mg/day. Its risk on human health was evaluated with THQ and it was less than 1. This indicates no potential non-carcinogenic harmful health risks to the consumers.

## 5 Recommendations

Despite the THQ < 1, this doesn’t mean safe. This value depends on the frequency of khat consumption and body weight of the consumer. Therefore, to reduce the health risk on the khat consumers, appropriate binding reclamations has to be developed to solve the challenges of land contamination with Cr from tannery effluents.

## Declarations

### Author contribution statement

Agegnehu Alemu: Conceived and designed the experiments; Performed the experiments; Analyzed and interpreted the data; Contributed reagents, materials, analysis tools or data; Wrote the paper.

Alemwork Mekonnen: Analyzed and interpreted the data; Wrote the paper.

### Funding statement

This research did not receive any specific grant from funding agencies in the public, commercial, or not-for-profit sectors.

### Data availability statement

Data included in article/supp. material/referenced in article.

### Declaration of interest’s statement

The authors declare no conflict of interest.

### Additional information

No additional information is available for this paper.
